# Goals and task difficulty expectations modulate striatal responses to feedback

**DOI:** 10.3758/s13415-014-0269-8

**Published:** 2014-03-18

**Authors:** Samantha DePasque Swanson, Elizabeth Tricomi

**Affiliations:** Department of Psychology, Rutgers University, Newark, NJ 07102 USA

**Keywords:** Basal ganglia, Motivation, Feedback, Reward

## Abstract

The striatum plays a critical role in learning from reward, and it has been implicated in learning from performance-related feedback as well. Positive and negative performance-related feedback is known to engage the striatum during learning by eliciting a response similar to the reinforcement signal for extrinsic rewards and punishments. Feedback is an important tool used to teach new skills and promote healthful lifestyle changes, so it is important to understand how motivational contexts can modulate its effectiveness at promoting learning. While it is known that striatal responses scale with subjective factors influencing the desirability of rewards, it is less clear how expectations and goals might modulate the striatal responses to cognitive feedback during learning. We used functional magnetic resonance imaging to investigate the effects of task difficulty expectations and achievement goals on feedback processing during learning. We found that individuals who scored high in normative goals, which reflect a desire to outperform other students academically, showed the strongest effects of our manipulation. High levels of normative goals were associated with greater performance gains and exaggerated striatal sensitivity to positive versus negative feedback during blocks that were expected to be more difficult. Our findings suggest that normative goals may enhance performance when difficulty expectations are high, while at the same time modulating the subjective value of feedback as processed in the striatum.

**Electronic supplementary material:**

The online version of this article (doi:10.3758/s13415-014-0269-8) contains supplementary material, which is available to authorized users.

Feedback about one’s performance is a valuable tool for facilitating learning. It is used by educators, mental health professionals, physicians, and others to teach new skills, encourage adaptive behaviors, and promote healthful lifestyle changes. However, the context in which feedback is received can influence how successfully it motivates learning. For example, negative feedback more effectively facilitates learning when individuals focus on increasing their knowledge, rather than on demonstrating their abilities (Cianci, Schaubroeck, & McGill, 2010), but is less effective when individuals are experiencing stereotype threat (fear of confirming a negative stereotype by performing poorly; Mangels, Good, Whiteman, Maniscalco, & Dweck, 2011).

Contextual factors that influence learning may do so through their effects on feedback processing in the striatum. As the input region of the basal ganglia, the striatum has been heavily implicated in reward processing and the motivation of reinforcement-driven behaviors (Balleine, Delgado, & Hikosaka, 2007; Robbins & Everitt, 1996; Shohamy, 2011). Activation in the striatum is greater following rewarding outcomes than following negative outcomes and appears to scale with prediction error, which is the discrepancy between expected and received rewards (O’Doherty, 2004; Schultz & Dickinson, 2000). During feedback-based learning, in which participants learn to make appropriate choices through trial and error, performance-related feedback engages the striatum in an analogous manner, even in the absence of extrinsic rewards (e.g., Daniel & Pollmann, 2010; Satterthwaite et al., 2012; Tricomi, Delgado, McCandliss, McClelland, & Fiez, 2006). Striatal responses to positive and negative outcomes are associated with learning to adapt behavior to maximize rewards (e.g., O’Doherty et al., 2004; Pessiglione, Seymour, Flandin, Dolan, & Frith, 2006; Schönberg, Daw, Joel, & O’Doherty, 2007), and proper functioning in this region is required for feedback- or reward-based learning, as evidenced by lesion studies and neuropsychology research (e.g., de Borchgrave, Rawlins, Dickinson, & Balleine, 2002; Shohamy et al., 2004). Due to its role in processing and learning from rewards, the striatum stands to play a critical role in the effects of motivation on feedback-based learning.

A region that modulates behavior on the basis of motivation should be sensitive to motivational context, and there is evidence for such sensitivity in the striatum. Striatal responses to rewards and punishments are modulated not only by objective stimulus properties, such as reward frequency, predictability, and magnitude, but also by subjective factors, including hunger/satiety, individual preferences, and the social contexts in which these outcomes are received (e.g., Delgado, Frank, & Phelps, 2005; Fliessbach et al., 2007; Hariri et al., 2006; Peters & Buchel, 2010.; Schultz, 2010; Tricomi, Rangel, Camerer, & O’Doherty, 2010). Because reward responses in the striatum are sensitive to such a variety of influences, the responses produced during feedback-based learning might be similarly modulated by an individual’s goals and expectations. Thus, the striatum may mediate the effects of achievement motivation on learning.

Goals and expectations for success are known to influence persistence, effort, and performance in achievement settings (Wigfield & Eccles, 2000). Expectations for success depend, in part, upon the perceived difficulty of the goal (Latham & Locke, 1991). In the present study, we manipulated beliefs about the difficulty of a novel feedback-based learning task, independently of actual task difficulty, to influence expectations for success. We aimed to explore the effects of these expectations on the motivational salience and instructive efficacy of feedback during learning. We predicted that feedback would engage the striatum and that beliefs about task difficulty would modulate striatal feedback responses during learning.

Because expectations about task difficulty may differentially impact individuals who vary in their goals, we further hypothesized that the effects of expectations on performance might depend critically upon individual differences in achievement goals. Achievement goals can be subdivided into performance versus learning/mastery goals (Elliott & Dweck, 1988), and performance goals can be further classified as either normative or ability goals (Grant & Dweck, 2003). Recent research suggests that those high in normative goals (e.g., “My goal in class is to get a better grade than most of the students”) fare better academically than those high in ability goals (e.g., “In school I am focused on demonstrating my intellectual ability”; Hulleman, Durik, Schweigert, & Harackiewicz, 2008; Hulleman, Schrager, Bodmann, & Harackiewicz, 2010).

Highly motivated individuals benefit from adopting competitive goals, and especially so when they are provided with information about the likelihood of performing well (Epstein & Harackiewicz, 1992). Thus, participants who spontaneously adopt normative goals, which are inherently competitive, may similarly benefit when they are provided with information about task difficulty. Individuals who are motivated by normative goals might exhibit enhanced interest and effort when they believe a task to be more difficult, since it would be more diagnostic of the differences between low- and high-performing individuals and would provide a chance for them to demonstrate their superiority. We expected that this might result in enhanced performance and exaggerated striatal responses to feedback during experimental blocks that are expected to be more difficult.

To investigate our hypotheses about the neural processing of cognitive feedback under varying levels of expected difficulty, we used functional magnetic resonance imaging (fMRI) during a feedback-based learning task. We hypothesized that striatal feedback responses would be stronger when the task instructions suggested a low probability of success (“HARD”-labeled blocks) and that individual differences in normative goals might moderate the relationship between expectations and feedback processing. Due to the relationship between striatal feedback responses and learning, we further hypothesized that striatal modulation by task difficulty expectations would be accompanied by enhanced performance on the learning task.

## Method

### Participants

Participants were recruited from the university community, were predominantly university students and staff, and possessed a broad range of demographics. Twenty right-handed adults (12 males), 18–35 years of age, completed the study. Four additional participants were excluded from analysis due to failure to finish the task (fatigue, *n* = 2; light-headedness, *n* = 1) and ceiling performance, which resulted in too few trials containing negative feedback (*n* = 1). All participants received compensation of $50 for their time spent in the experiment. Our procedures were approved by the institutional review boards of Rutgers University and the University of Medicine & Dentistry of New Jersey (UMDNJ).

### Materials and procedure

#### Experimental task

**Fig. 1:**
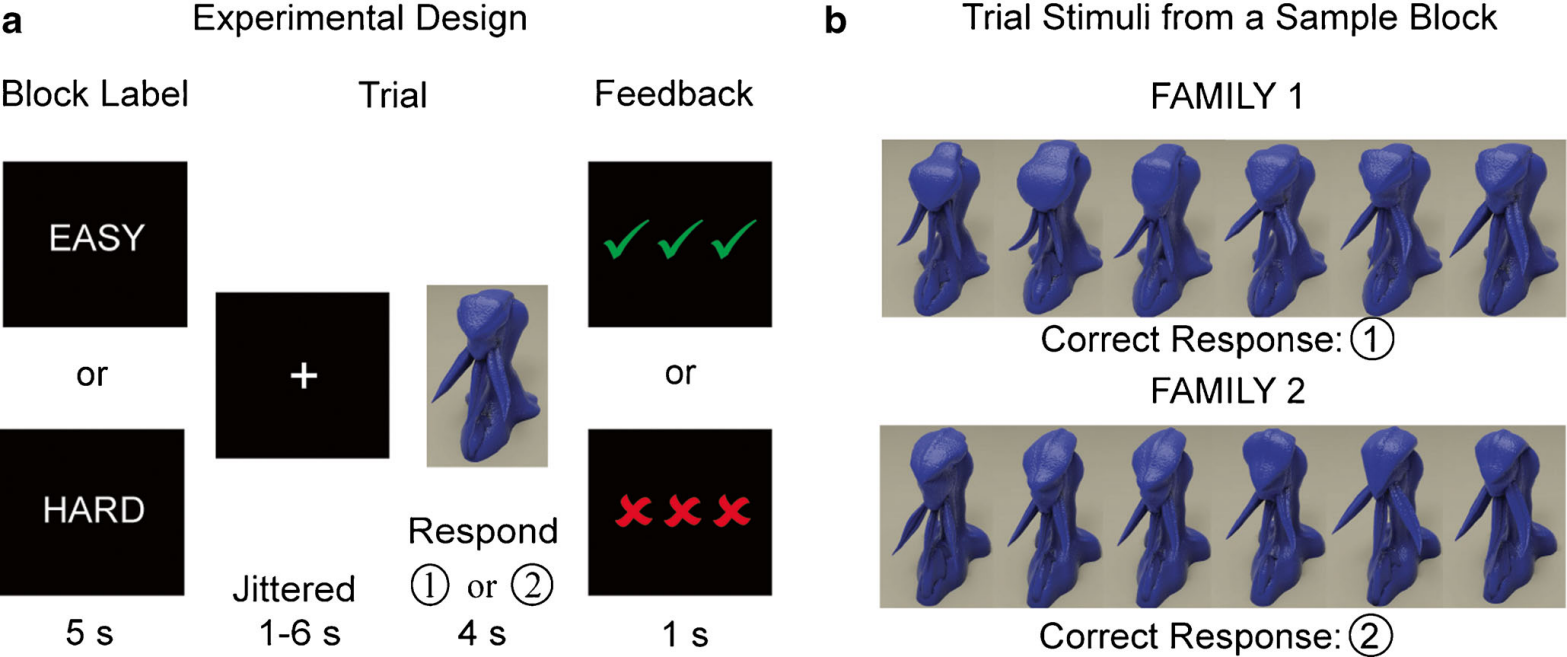
Experimental design. **a** Sixteen blocks of trials were each preceded by an arbitrary task difficulty label, presented to influence expectations about task difficulty. Twelve trials per block included a jittered fixation (1–6 s), stimulus presentation/response period (4 s), and immediate presentation of positive or negative feedback (1 s). **b** Within each block of trials, 12 distinct figures from two different families would appear in random order. Members of one family were associated with the first button on the button box, while members of the second family were associated with the second button. During the next block, members from two new families would be randomly presented

We developed a novel visual categorization learning task with arbitrary block difficulty labels, presented in a mixed-block/event-related design (see Fig. 1a). Participants learned to categorize figures from eight different “families” of alien-like creatures through trial-and-error responding with feedback (stimulus images courtesy of Michael J. Tarr, Center for the Neural Basis of Cognition and Department of Psychology, Carnegie Mellon University, http://www.tarrlab.org).

Sixteen blocks of trials contained 12 trials each, with 12 distinct stimuli in each block sampled from two of the eight families (Fig. 1b). During each trial, a single figure appeared on the screen for 4 s. During stimulus presentation, the participant made a judgment about the family membership of the figure by pressing one of two buttons on an MRI-compatible button box. Each of the 12 figures within a given block belonged to one of two families; six members of one family were associated with one button, and members of the other family were associated with the alternate button. Feedback was presented for 1 s, immediately after the 4-s stimulus screen: Correct responses resulted in green checkmarks (**√√√**), incorrect responses resulted in red “X”s (XXX), and no response resulted in three dashes (---). A jittered fixation cross appeared for 1–6 s following the feedback. We did not include jitter between the stimulus screen and feedback, since previous research suggests that delaying feedback by even a few seconds can influence learning strategies and diminish striatal responsiveness to feedback (e.g., Foerde & Shohamy, 2011; Maddox, Ashby, & Bohil, 2003).

#### Stimuli

The stimuli used in this study (“YUFOs”) come from a set of rendered 3-D objects used previously in vision research, which are highly visually similar, sharing the same size, color, and general spatial configuration (Gauthier, James, Curby, & Tarr, 2003; Rossion, Kung, & Tarr, 2004). Subtle differences in shape can be used to distinguish stimuli from different families. Within each family, the stimuli were all variations on the same basic shape, and pilot testing demonstrated that participants were able to learn to discriminate between stimuli from the different families at above-chance levels. Within families, there were “male” and “female” figures with differences in shape that were consistent across families. To create blocks of trials that were objectively easier, we selected stimuli that differed both in family and in gender, as opposed to stimuli that differed only in family. Because the differences in shape do not tend to be simple or easily verbalizable, a rule-based strategy is not ideal for performing well on the task.

#### Manipulation

There were two levels of actual difficulty (high and low), based on the visual similarity of the two families in each block, crossed with two levels of labeled difficulty (labeled “HARD” and “EASY”). Task difficulty labels appeared at the beginning of each new block, for 5 s before the trials began. A four-block training session preceded the 16 experimental blocks. Instructions emphasized that the participant’s goal was to learn which aliens come from which families and that sometimes the differences between families could be very subtle and, therefore, harder to tell apart. Participants were informed that the blocks that contained families with very subtle differences would be labeled “HARD,” while the others would be labeled “EASY.” During training, the labeled difficulty always matched the actual difficulty level, to strengthen the expectation that an “EASY” block would be easier to perform than a “HARD” block. During the 16 experimental blocks, the difficulty labels were independent of the true difficulty level but were presented to influence task difficulty expectations. The 16 experimental blocks were evenly divided across the four conditions (low difficulty, labeled “EASY”; low difficulty, labeled “HARD”; high difficulty, labeled “EASY”; high difficulty, labeled “HARD”).

#### Questionnaires

After the training session but before the experimental trials, participants rated their perception of the difference between the (objectively more difficult) “HARD” blocks and the “EASY” blocks, on a scale of 1 (*certain there was a difference*) to 4 (*certain there was no difference*). After the conclusion of the fMRI study, each participant completed a postexperiment questionnaire to determine whether they continued to believe in the difficulty labels throughout the task. The open-ended responses were coded according to whether participants expressed suspicion in the accuracy of the labels (1) or not (0). In addition, participants completed the Achievement Goal Inventory (Grant & Dweck, 2003), an 18-item questionnaire that distinguishes between normative goals (6 items, α = .92—e.g., “My goal in class is to get a better grade than most of the students” ) and nonnormative ability goals (3 items, α = .81—e.g., “In school I am focused on demonstrating my intellectual ability”). Additional subscales include learning goals (6 items, α = .86) and outcome goals (3 items, α = .85). Agreement with each statement was rated on a Likert scale from 1 (*strongly disagree*) to 7 (*strongly agree*), and responses to individual subscales were averaged to produce a single score for each. Analyses in the present study focused on the normative and ability subscales in particular.

### Data analysis

#### Behavioral analysis

Task performance was defined as the percentage of trials with correct responses in each condition. Two within-subjects factors (actual difficulty and labeled difficulty) were used in a 2 × 2 analysis of variance (ANOVA) to assess the effect of labeled difficulty on performance and whether the effect differed depending on actual difficulty. *T*-tests were used to test significance of the labeled difficulty effect within each level of actual difficulty. To determine whether normative goals might modulate the effect of expectations on performance, individual differences in the magnitude of the expectation effect were calculated by subtracting performance (% correct) on “EASY” trials from performance on “HARD” trials, separately for low-difficulty and high-difficulty blocks. For the conditions under which an expectation effect was observed, the magnitude of the effect was entered into bivariate correlations with normative goals and ability goals.

#### fMRI data collection and analysis

Scanning took place at the UMDNJ Advanced Imaging Center, with a 3 Tesla Siemens Allegra scanner and standard eight-channel head coil. Stimulus presentation and behavioral data collection were implemented with E-Prime software (Psychology Software Tools, Pittsburgh, PA). The fMRI data were preprocessed and analyzed using BrainVoyager software version 2.3.1 (Brain Innovation, Maastricht, The Netherlands). Preprocessing included motion correction, spatial smoothing (8 mm, FWHM), and high-pass temporal filtering. Preprocessed data were spatially normalized to the Talairach stereotaxic space (Talairach & Tournoux, 1988). After preprocessing, the Talairach-transformed fMRI data were analyzed using a random-effects general linear model (GLM) that focused on activation at the time of feedback presentation. The predictors of interest were modeled as events at the time of feedback onset and convolved with a canonical hemodynamic response function. These predictors included positive and negative feedback during each of the four experimental conditions: low versus high actual difficulty crossed with “EASY” versus “HARD” difficulty label. In addition, the model included the onset of the difficulty labels that occurred at the start of each block. Missed trials and six motion parameters were included in the model as predictors of no interest.

Due to our a priori interest in feedback responses within the striatum, we examined feedback-related activation in three bilateral regions of interest (ROIs), created by drawing five-mm spheres centered around coordinates in left and right caudate nucleus (±12, 8, 11), putamen (±24, 4, 3), and ventral striatum (±12, 7, −7) and combining the left and right spheres from each subregion into a single ROI. These coordinates were selected because they represent each of the major subdivisions within the striatum and were converted to Talairach coordinates from MNI coordinates that have been used in previous literature (e.g., Zink. Pagnoni, Martin, Dhamala, & Berns, 2003). In our data, the overall patterns of activation observed within these ROIs did not differ between the left and right hemispheres, so we report the results from each of the three combined bilateral ROIs. To explore effects of actual difficulty, instructed difficulty, and feedback valence in the striatum, beta estimates from each ROI were subjected to a 2 (actual difficulty) × 2 (labeled difficulty) × 2 (feedback valence) repeated measures ANOVA. We also performed a bivariate correlation for each ROI, between normative goals and the effect of expectations on feedback sensitivity: “HARD” (positive > negative feedback) > “EASY” (positive > negative feedback). To determine whether these regions demonstrated any differential response at the time of label onset, we subjected the parameter estimates from each ROI to a *t*-test comparing activation during the onset of the “EASY” labels to activation at the onset of the “HARD” labels. Whole-brain analyses were also conducted as detailed in the Supplemental Methods.

A second random-effects GLM was also used to explore condition-related differences in sustained activation during the entire duration of each block. In this second GLM, the entire duration of each block was modeled as an epoch, from the onset of the first trial to the offset of the last trial. The ROI beta estimates were subjected to a 2 (actual difficulty) × 2 (labeled difficulty) ANOVA to determine whether sustained activation differed as a function of actual difficulty, labeled difficulty, or an interaction between the two factors.

## Results

### Behavioral results

**Fig. 2:**
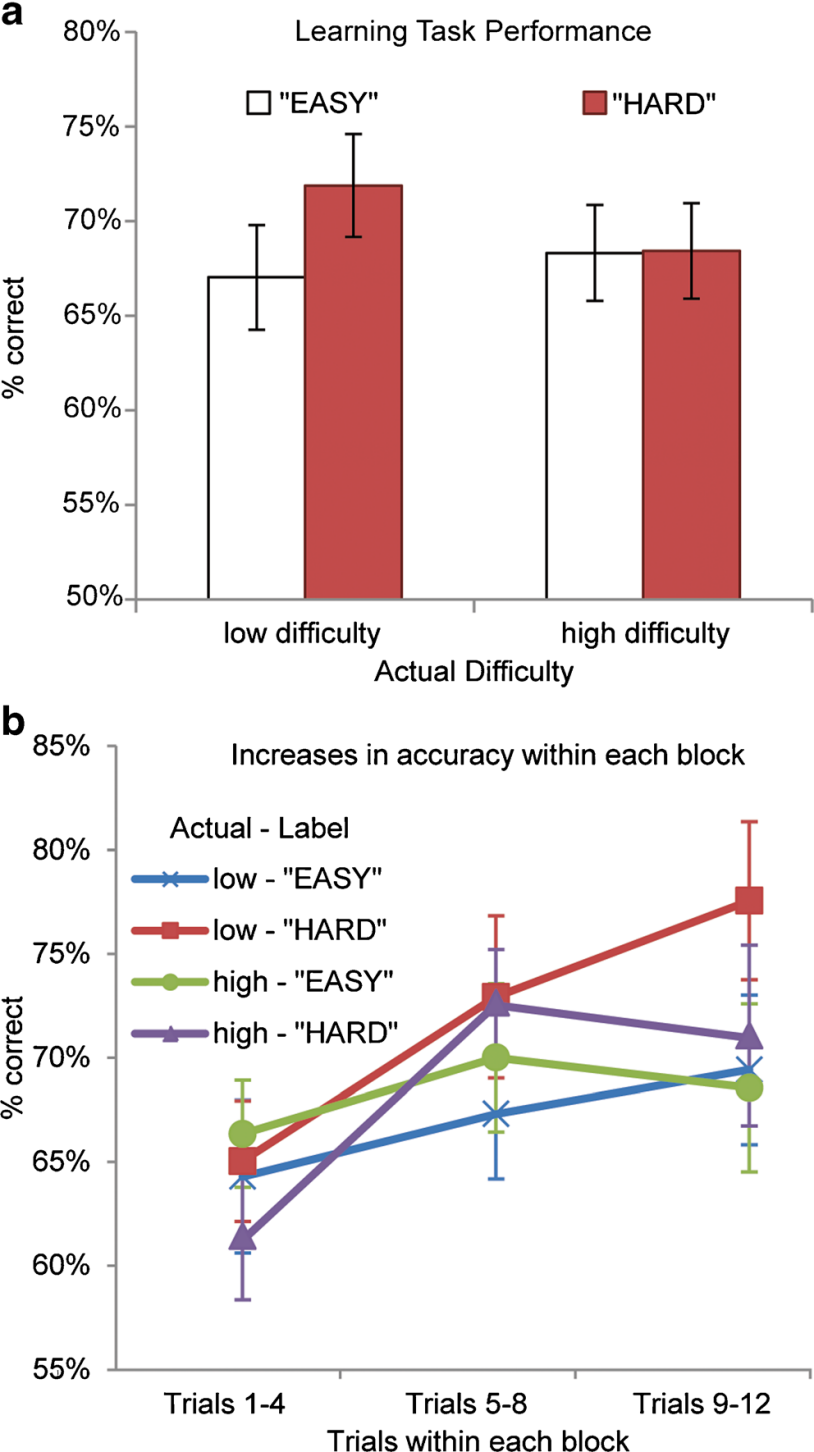
Behavioral results. **a** For low-difficulty blocks, performance was significantly better for “HARD”-labeled than for “EASY”-labeled blocks. **b** Percent correct responses are plotted for early trials (trials 1–4), middle trials (5–8), and late trials (9–12) within each block, to visualize the time course over which expectations influenced learning. Expectation-related performance differences emerged during the late trials for low-difficulty blocks

Overall, participants were able to perform above chance on the task (M = 68.75 % correct, *SD* = 9.63 %), with a wide range of scores (min = 52.88 %, max = 87.50 %) suggesting diverse ability levels. Participants exhibited a broad range of scores on both the normative and ability goal subscales of the Achievement Goal Inventory (normative *M* = 3.625, *SD* = 1.107, min = 1, max = 5; ability *M* = 4.367, *SD* = 1.048, min = 1.333, max = 6). No gender differences were observed in measures of performance or achievement goals. A 2 × 2 repeated measures ANOVA detected no main effects of either labeled difficulty (“EASY” vs. “HARD”) or actual difficulty (low vs. high) on task performance, although a trend emerged toward an interaction of labeled difficulty and actual difficulty, *F* = 3.54, *p* = .075. As can be seen in Fig. 2a, there was a significant effect of labeled difficulty for the blocks that were low in actual difficulty, where performance was superior during “HARD”-labeled blocks, *t*(19) = 2.17, *p* = .043 (two-tailed). No such difference was observed for the high-difficulty blocks, *t*(19) < 0.01, *p* = .997 (two-tailed). The performance differences for low-difficulty blocks emerged during the final trials of each block, as depicted in the Fig. 2b. *T*-tests that focused on the percentage of correct responses during trials 9–12 exhibited the same pattern of results as the original analysis: For low-difficulty blocks, participants performed significantly better on “HARD”- than “EASY”-labeled blocks, t(19) = 2.303, *p* = .033, but for high-difficulty blocks, the same pattern did not hold, *t* = 0.570, *p* = .575.

**Fig. 3:**
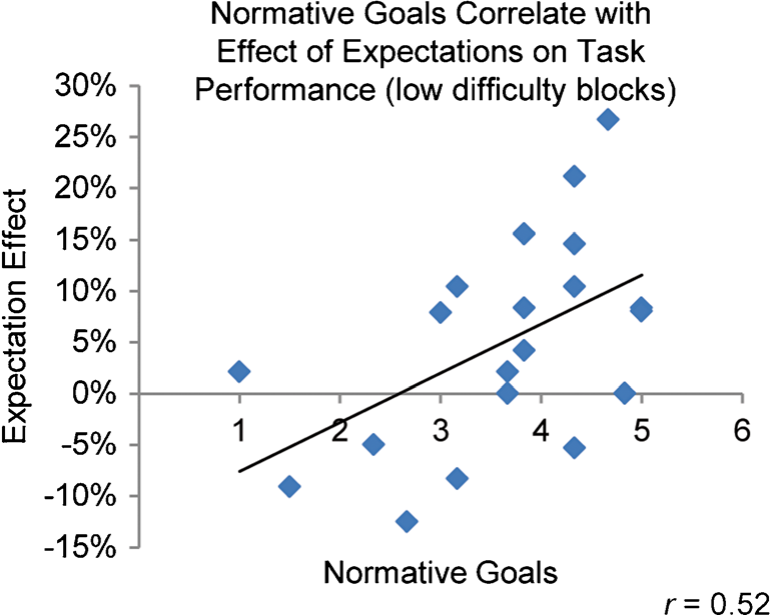
Correlation between normative goals and expectation effect. Average scores from the normative goals subscale of the Achievement Goal Inventory were positively correlated with the effect of expectations on task performance for low-difficulty blocks, *r*(18) = .52, *p* = .019. Expectation effect is defined as the difference in proportion of correct responses on the “HARD”-labeled blocks versus “EASY”-labeled blocks (proportion correct “HARD” −proportion correct “EASY”)

To assess individual differences in the effects of expectations on learning, an “expectation effect” for low-difficulty blocks was calculated for each participant by subtracting percent correct on the “EASY”-labeled subset of those blocks from percent correct on the “HARD”-labeled subset. Expectation effects ranged from −.10 to .27 (*M* = .05, *SD* = .10), with positive values indicating better performance on “HARD”-labeled blocks and negative values indicating better performance on “EASY”-labeled blocks. As is displayed in Fig. 3, the size of the expectation effect was positively correlated with the normative goals subscale of the achievement goal questionnaire, *r*(18) = .52, *p* = .019. Participants who expressed higher levels of normative goals showed greater performance benefits from expectations of higher difficulty, specifically for the low-difficulty blocks in which high performance was objectively more attainable. Despite this increased tendency to perform better on “HARD” than on “EASY” blocks, normative goals were not correlated with overall task performance, *r*(18) = −.06, *p* = .817, suggesting that the effect of normative goals on performance was related to the effect of expectations, rather than baseline ability levels. In contrast to normative goals, ability goals did not exhibit a relationship with the effect of expectations on performance, *r*(18) = −.144, *p* = .544.

After training, the majority of participants reported perceiving the difference in difficulty between “EASY” and “HARD” blocks (*n* = 15). However, after the conclusion of the experiment, many participants reported suspecting a possible mismatch between the label and the actual difficulty (*n* = 15). Among the participants for whom training questionnaire data were available (data for 1 participant not logged, due to software malfunction), ratings indicating greater perceived difference between “EASY”- and “HARD”-labeled blocks during the practice session (when labels were accurate) were negatively correlated with the tendency to suspect that the labels were false at the end of the study, *r*(17) = −.518, *p* = .023. Given that we observed an effect of expectations on performance, it is likely that for many, the mismatch did not become apparent until late in the experiment or when filling out the questionnaire.

### fMRI results

**Fig. 4:**
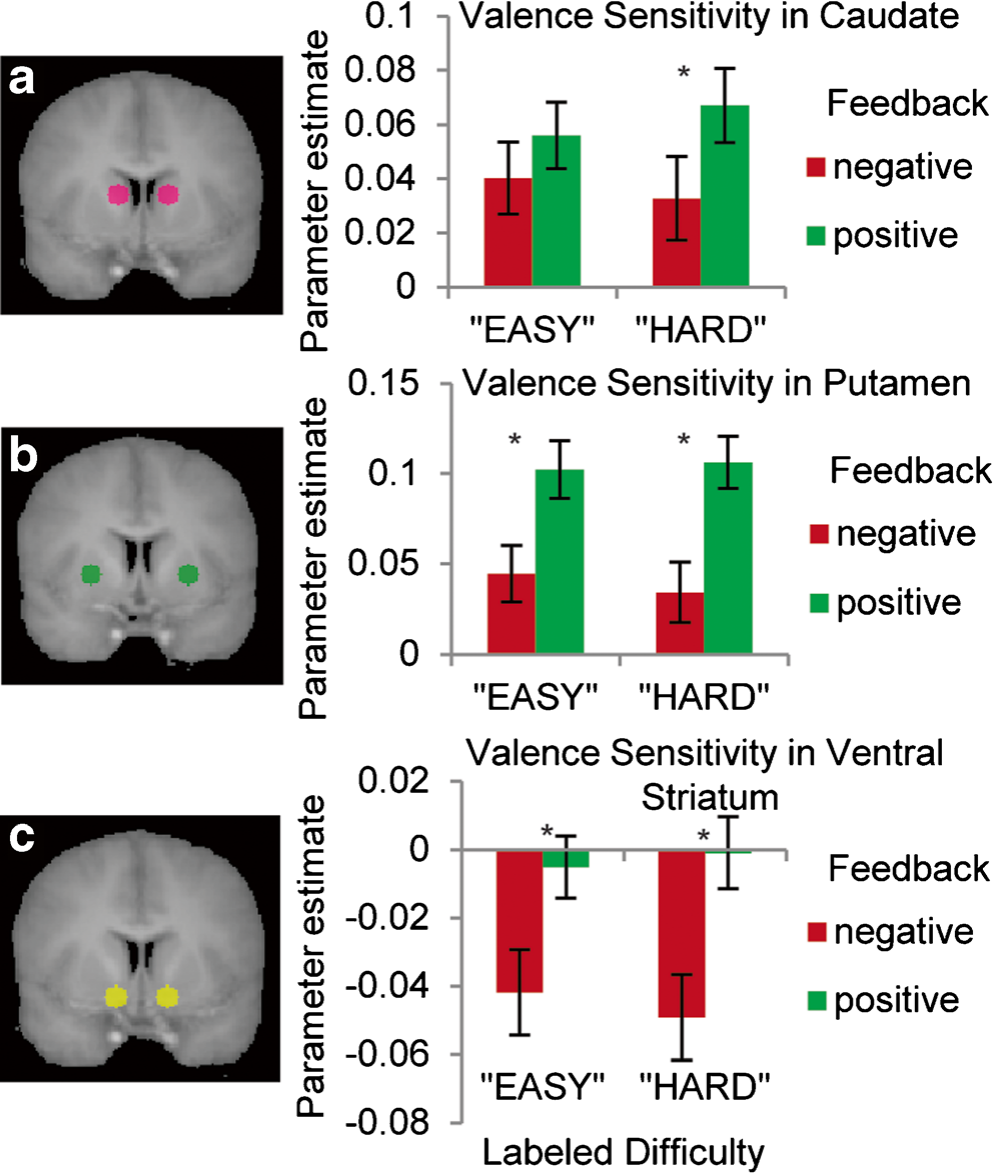
Valence sensitivity in “EASY”- and “HARD”-labeled blocks. Positive feedback elicited greater activation than did negative feedback in each of the striatal regions of interest (ROIs). **a** The caudate ROI reliably distinguishes between positive and negative feedback during “HARD”-labeled blocks, *t*(19) = 2.618, *p* = .017, but not during “EASY”-labeled blocks, *t*(19) = 1.465, *p* = .159). **b** Feedback activation in the putamen. **c** Feedback activation in the ventral striatum

The results of a 2 × 2 × 2 repeated measures ANOVA are reported for each ROI in Table 1. As was predicted, feedback valence modulated activation in each of the striatal ROIs. In the caudate, putamen, and ventral striatum, activation at the onset of positive feedback exceeded that for negative feedback across all task conditions (see Fig. 4). Supplemental Table 1 lists valence-sensitive regions identified by the whole-brain GLM analysis, including peaks within the putamen and ventral striatum. No other main effects from the event-related GLM reached significance in the three striatal ROIs (see Supplemental Tables 2–4 for regions outside the striatum exhibiting effects of actual difficulty, labeled difficulty, and the interaction of labeled difficulty and feedback valence). However, several analyses identified trends that suggest that null findings should be interpreted with caution. A medium effect size was observed for the main effect of actual difficulty in the putamen, *F* = 2.31, *p* = .146, *η* ^2^_p_ = .108, with greater feedback-related activation for high-difficulty blocks than for low-difficulty blocks. An interaction between actual difficulty and feedback valence also exhibited a medium effect size in the caudate, *F* = 2.872, *p* = .106, *η* ^2^_p_ = .131, with greater differentiation between positive and negative feedback during high- than low-difficulty trials.

**Table 1:** Results of the 2 × 2 × 2 repeated measures ANOVA in three a priori striatal regions of interest

Region of Interest	Direction of Effect	*F* Value	*p* Value	*η* ^2^_p_	Effect Size
Main Effects					
Actual difficulty					
Caudate nucleus	High > Low	0.77	.391	.039	Small
Putamen	High > Low	2.31	.146	.108	Medium
Ventral striatum	Low > High	0.265	.613	.014	Small
Labeled difficulty					
Caudate nucleus	–	0.032	.859	.002	–
Putamen	–	0.109	.744	.006	–
Ventral striatum	–	0.057	.814	.003	–
Valence					
Caudate nucleus	Positive > Negative	7.56*	.013	.285	Large
Putamen	Positive > Negative	46.743*	.000	.711	Large
Ventral striatum	Positive > Negative	19.695*	.000	.509	Large
Interactions					
Actual difficulty × labeled difficulty					
Caudate nucleus	--	0.009	.925	.000	–
Putamen	--	0.001	.978	.000	–
Ventral striatum	Congruent label > Incongruent label	0.353	.559	.018	Small
Actual difficulty × valence					
Caudate nucleus	High (pos > neg) > Low (pos > neg)	2.872	.106	.131	Medium
Putamen	--	0.079	.781	.004	–
Ventral striatum	High (pos > neg) > Low (pos > neg)	0.651	.43	.033	Small
Label × valence					
Caudate nucleus	“HARD” (pos > neg) > “EASY” (pos > neg)	1.412	.249	.069	Medium
Putamen	“HARD” (pos > neg) > “EASY” (pos > neg)	0.583	.455	.03	Small
Ventral striatum	“HARD” (pos > neg) > “EASY” (pos > neg)	1.205	.286	.06	Small

Most notably, both caudate and ventral striatum demonstrated medium effect sizes for the interaction between label and valence, such that the differentiation between positive and negative feedback was greater during “HARD”-labeled blocks than during “EASY”-labeled blocks (caudate, *F* = 1.412, *p* = .249, *η* ^2^_p_ = .069, Fig. 4a; ventral striatum, *F* = 1.205, *p* = .286, *η* ^2^_p_ = .06, Fig. 4c). This interaction is most evident in the caudate, where differentiation between positive and negative feedback is only significant in “HARD”-labeled blocks (see Fig. 4a). Although these effects were nonsignificant, each of the trends reported above can be characterized as medium effect sizes according to the guidelines set forth by Cohen (1988) and may have reached significance in a study with greater power to detect subtle effects. Supplemental Fig. 1 illustrates the broader extent of activation observed throughout the brain for positive > negative feedback during “HARD”-labeled blocks relative to “EASY”-labeled blocks, which is again consistent with the idea that striatal valence sensitivity may be modulated by expectations about task difficulty.

**Fig. 5:**
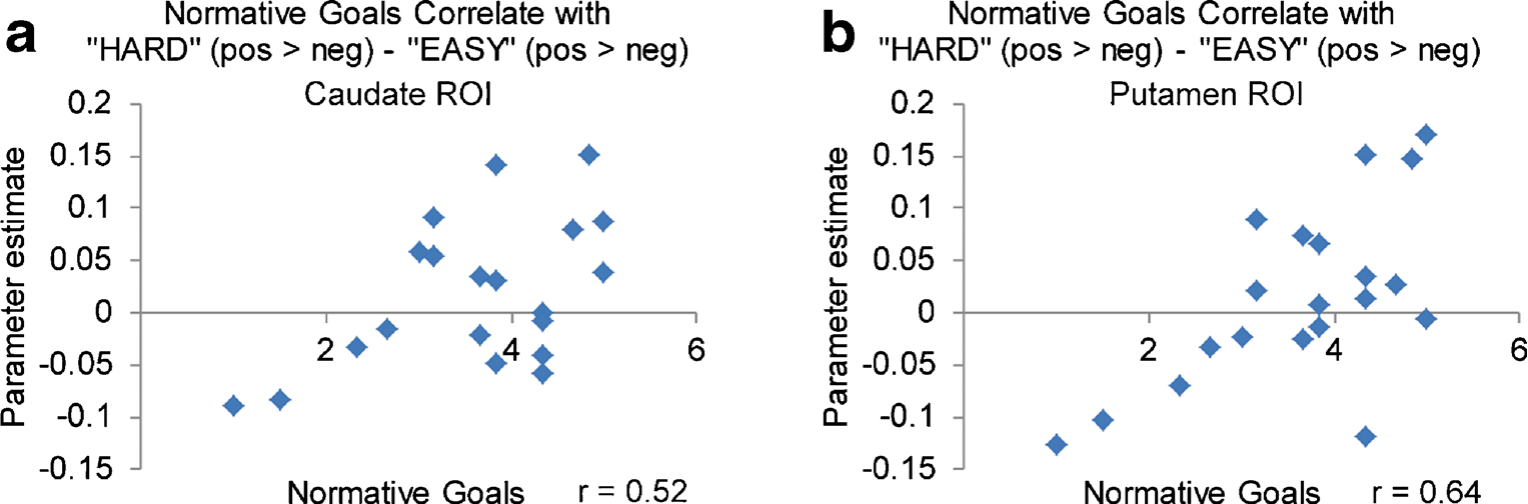
Correlations between normative goals and expectation by valence interaction. In the caudate (**a**) and putamen (**b**), normative goals were significantly positively correlated with the difference in “HARD” positive > negative feedback processing versus “EASY” positive > negative feedback processing

Normative goals exhibited a significant correlation with the effect of labeled difficulty on feedback valence sensitivity (“HARD” positive vs. negative feedback > “EASY” positive vs. negative feedback) in both the caudate, *r* = .518, *p* = .019 (Fig. 5a), and the putamen, *r* = .635, *p* = .003 (Fig. 5b). These ROI results are corroborated by a whole-brain ANCOVA, which identified a region in the putamen in which normative goals correlated with the effect of expectations on valence sensitivity (see Supplemental Fig. 2). This relationship suggests that those individuals who are most motivated to outperform their peers exhibit the strongest effect of expectations on feedback processing in the dorsal striatum.

The behavioral expectation effect (% correct trials in low-difficulty “EASY” versus “HARD” blocks) was not significantly correlated with the effect of expectations on valence sensitivity in the three striatum ROIs (caudate, *r*(18) = .258, *p* = .272; putamen, *r*(18) = .209, *p* = .376; ventral striatum, *r*(18) = .052, *p* = .828). None of the striatal ROIs exhibited differential activation at the time that the difficulty label was displayed to start each block or any significant differences in sustained activation throughout each block as a function of actual or labeled difficulty. However, a whole-brain analysis identified some regions that exhibited a main effect of actual difficulty on sustained activation, including a cluster in the putamen (Supplemental Table 5).

## Discussion

The goal of this study was to investigate whether feedback-related activation in the striatum is sensitive to subjective expectations and goals. We have shown that task difficulty expectations modulate striatal sensitivity to positive versus negative feedback for individuals with high normative goals. That is, the beneficial effects of increased task difficulty expectations were most pronounced for these participants. This suggests that the desire to perform well in comparison with one’s peers may influence the affective response to task difficulty expectations and enhance learning for low-difficulty tasks that are expected to be difficult. The behavioral effect may have been absent for high-difficulty blocks because performance was objectively more difficult to improve. Thus, motivation may have been affected for both low- and high-difficulty blocks, but enhanced task investment may have paid off only in blocks in which the correct categories were more easily discerned.

### Achievement goals and feedback sensitivity

The effect of normative goals on task performance was reflected in feedback processing in the striatum. Specifically, in the caudate and putamen, normative goals were positively correlated with a larger effect of expectations on striatal sensitivity to feedback valence. Such striatal differentiation between positive and negative feedback has been previously associated with the ability to learn from trial and error (e.g., O’Doherty et al., 2004; Pessiglione et al., 2006; Schönberg et al., 2007). Thus, because the differentiation we observed was greater in “HARD”-labeled blocks than in “EASY”-labeled blocks for participants who were highest in normative goals, it is fitting that these same participants also showed the greatest performance benefits from being told a low-difficulty block would be “HARD.”

Given these results, we suggest that a desire to measure up favorably against other participants may result in a greater commitment to performing well in the “HARD” blocks. Under these circumstances, participants might value positive feedback more strongly and find negative feedback more aversive, due to the greater affective investment in performing well. Without subjective ratings of the importance of performing well on “HARD” versus “EASY” blocks, this remains speculative. However, this interpretation is consistent with our finding of a correlation between normative goals and heightened striatal sensitivity to feedback valence during blocks that are expected to be more difficult.

### Feedback processing in the striatum

As was anticipated, positive feedback resulted in greater activation in the striatum, as compared with negative feedback. The valence effects we observed in the ventral striatum are consistent with previous studies of reward learning and prediction error, where positive outcomes result in greater activation in the nucleus accumbens and ventral putamen (Breiter, Aharon, Kahneman, Dale, & Shizgal, 2001; McClure, Berns, & Montague, 2003; O’Doherty et al., 2004). However, while previous studies have found cognitive feedback responses in the head of the caudate (e.g., Daniel & Pollmann, 2010; Dobryakova & Tricomi, 2013; Tricomi et al., 2006), the present task also produced activation in the putamen. Valence sensitivity has been previously observed in the putamen during reward learning (e.g., Delgado, 2007; Liu, Hairston, Schrier, & Fan, 2011; Luking & Barch, 2014), but the key factors that determine which striatal subregions will be activated for a particular task are still being explored (Lopez-Paniagua & Seger, 2011). Future research will be needed to further clarify the roles of striatal subregions in different types of feedback-based learning tasks.

### Relation to prior research

The finding that normative goals enhanced the effect of task difficulty expectations on learning complements the growing body of evidence that performance goals can be beneficial when they are normative in nature, because they can prompt individuals to set higher standards for themselves and help them excel when they perceive those goals as achievable (Hulleman et al., 2008; Hulleman et al., 2010). Superior exam performance for those high in normative goals has been attributed to increased effort and persistence (Elliot, McGregor, & Gable, 1999), so it is possible that participants in our study who adopted normative goals may have invested more effort during blocks they expected to be more challenging. The amount of effort exerted during a task can influence striatal sensitivity to rewards and losses (Hernandez Lallement et al., 2014); thus, our finding of enhanced striatal sensitivity during “HARD”-expected blocks for those high in normative goals may be due to enhanced effort during those blocks. It is also possible, since striatal feedback activation reflects goal satisfaction (Han, Huettel, Raposo, Adcock, & Dobbins, 2009), that the modulation of activation in our task is caused by enhanced motivation affecting the subjective value of performing well during those blocks. This interpretation is consistent with a large body of research that suggests that reward responses in the striatum vary with the subjective value of outcomes (Bartra, McGuire, & Kable, 2013). Furthermore, normative goals are inherently competitive, and social competition has been shown to increase the amount people are willing to pay at auctions (e.g., Delgado, Schotter, Ozbay, & Phelps, 2008; Goeree, Holt, & Palfrey, 2002). If willingness to pay is viewed as a proxy for subjective value, then there is evidence that competitive goals can drive subjective value. Future research will be needed to tease apart the effects of effort per se and the enhanced motivation that may occur for individuals high in normative goals when a task is expected to be more challenging.

Our finding that achievement goals and contextual information can jointly influence striatal sensitivity to feedback during learning fits within the broader picture of research showing modulation of striatal reward responses by individual differences in motivation and goals. For instance, individual differences in reward sensitivity, drive, extrinsic versus intrinsic motivational orientation, and trait approach versus avoidance motivation have been found to influence responses to rewarding and aversive stimuli in the ventral striatum, putamen, and caudate (Beaver, Lawrence, Passamonti, & Calder, 2008; Costumero et al., 2013; Linke et al., 2010; Spielberg et al., 2012). Our results extend this work by demonstrating that striatal processing of cognitive feedback is sensitive to variation in expectations and goals.

### Limitations

Our study demonstrated a modulatory effect of normative goals on the influence of task difficulty expectations on both performance and striatal sensitivity to positive versus negative feedback. However, in order to firmly establish the effects of the difficulty labels on participants’ expectations, we used training blocks that differed from the experimental task in that they used only veridical task difficulty labels. To avoid confounding effects of actual difficulty with effects of expectations, by necessity, half of the blocks in the experimental task contained difficulty labels that were false. It is possible that presenting only accurately labeled blocks during training could have influenced subsequent performance by providing an opportunity for participants to learn subtle differences in strategy that could distinguish low- from high-difficulty blocks and, thus, reduce their belief in the labels during the experiment. However, pilot testing of the experimental paradigm suggested that participants were more likely to believe in the manipulation if the difficulty levels of the easy and the hard blocks were experienced as noticeably different during training. Data from our fMRI participants are consistent with this view, in that the participants who did not notice a difference in difficulty during training appeared to be the most likely to report suspicion about the difficulty labels after the study concluded. If we had included training blocks with a mix of accurate and inaccurate difficulty labels, participants may have learned even sooner that the difficulty labels did not appear to reflect actual task difficulty, and thus our results may not have been as strong.

An additional limitation of the present study is that it did not demonstrate a direct relationship between striatal sensitivity to feedback valence and performance on the learning task. One possibility is that this could reflect the declarative nature of the task. Although the striatum is engaged by positive and negative feedback in this task, it is possible that the magnitude of the striatal response is not as directly responsible for declarative learning as it is for nondeclarative learning (e.g., Poldrack et al., 2001). The modulation of feedback responses by normative goals may have more strongly reflected the motivational salience of the feedback than the amount learned from the feedback. Further research will be needed to determine the precise neural mediators of the effects of normative goals on learning.

### Conclusion

This study provides novel insight into a potential neural mechanism for the interaction between trait academic achievement motivation and contextual influences on learning. The integration of the psychology of academic achievement motivation and the neuroscience of feedback-based learning allowed us to probe the joint effects of personality and context on the striatal processing of performance-related feedback. Due to the relationship we observed between normative goals, task difficulty expectations, and the magnitude of the striatal response to positive and negative feedback, we suggest that striatal feedback responses are influenced by the affective salience of the feedback. We have demonstrated that striatal processing of cognitive feedback can be affected by goals and expectations, much like the modulation of extrinsic reward responses by subjective value. These results lend support to the notion that the striatum is a key region in the modulation of learning by achievement motivation.

## Electronic supplementary material

Below is the link to the electronic supplementary material.

ESM 1 (PDF 128 kb)

## References

[CR1] Balleine BW, Delgado MR, Hikosaka O (2007). The role of the dorsal striatum in reward and decision-making. Journal of Neuroscience.

[CR2] Bartra O, McGuire JT, Kable JW (2013). The valuation system: A coordinate-based meta-analysis of BOLD fMRI experiments examining neural correlates of subjective value. NeuroImage.

[CR3] Beaver JD, Lawrence AD, Passamonti L, Calder AJ (2008). Appetitive motivation predicts the neural response to facial signals of aggression. Journal of Neuroscience.

[CR4] Breiter HC, Aharon I, Kahneman D, Dale A, Shizgal P (2001). Functional imaging of neural responses to expectancy and experience of monetary gains and losses. Neuron.

[CR5] Cianci AM, Schaubroeck JM, McGill GA (2010). Achievement goals, feedback, and task performance. Human Performance.

[CR6] Cohen J (1988). Statistical power analysis for the behavioral sciences.

[CR7] Costumero, V., Barrós-Loscertales, A., Bustamante, J. C., Ventura-Campos, N., Fuentes, P., & Avila, C. (2013). Reward sensitivity modulates connectivity among reward brain areas during processing of anticipatory reward cues. *The European Journal of Neuroscience*, (January), 1–9. 10.1111/ejn.12234 23617942

[CR8] Daniel R, Pollmann S (2010). Comparing the neural basis of monetary reward and cognitive feedback during information-integration category learning. Journal of Neuroscience.

[CR9] De Borchgrave R, Rawlins JN, Dickinson A, Balleine BW (2002). Effects of cytotoxic nucleus accumbens lesions on instrumental conditioning in rats. Experimental Brain Research.

[CR10] Delgado MR (2007). Reward-related responses in the human striatum. Annals of the New York Academy of Sciences.

[CR11] Delgado MR, Frank RH, Phelps EA (2005). Perceptions of moral character modulate the neural systems of reward during the trust game. Nature Neuroscience.

[CR12] Delgado MR, Schotter A, Ozbay EY, Phelps EA (2008). Understanding overbidding: Using the neural circuitry of reward to design economic auctions. Science.

[CR13] Dobryakova E, Tricomi E (2013). Basal ganglia engagement during feedback processing after a substantial delay. Cognitive, Affective & Behavioral Neuroscience.

[CR14] Elliot AJ, McGregor HA, Gable S (1999). Achievement goals, study strategies, and exam performance: A mediational analysis. Journal of Educational Psychology.

[CR15] Elliott ES, Dweck CS (1988). Goals: An approach to motivation and achievement. Journal of Personality and Social Psychology.

[CR16] Epstein JA, Harackiewicz JM (1992). Winning is not enough: The effects of competition and achievement orientation on intrinsic interest. Personality and Social Psychology Bulletin.

[CR17] Fliessbach K, Weber B, Trautner P, Dohmen T, Sunde U, Elger CE, Falk A (2007). Social comparison affects reward-related brain activity in the human ventral striatum. Science.

[CR18] Foerde K, Shohamy D (2011). Feedback timing modulates brain systems for learning in humans. Journal of Neuroscience.

[CR19] Gauthier I, James TW, Curby KM, Tarr MJ (2003). The influence of conceptual knowledge on visual discrimination. Cognitive Neuropsychology.

[CR20] Goeree JK, Holt CA, Palfrey TR (2002). Quantal response equilibrium and overbidding in private-value auctions. Journal of Economic Theory.

[CR21] Grant H, Dweck CS (2003). Clarifying achievement goals and their impact. Journal of Personality and Social Psychology.

[CR22] Han S, Huettel SA, Raposo A, Adcock RA, Dobbins IG (2009). Functional significance of striatal responses during episodic decisions: Recovery or goal attainment?. Journal of Neuroscience.

[CR23] Hariri AR, Brown SM, Williamson DE, Flory JD, de Wit H, Manuck SB (2006). Preference for immediate over delayed rewards is associated with magnitude of ventral striatal activity. Journal of Neuroscience.

[CR24] Hernandez Lallement, J., Kuss, K., Trautner, P., Weber, B., Falk, A., & Fliessbach, K. (2014). Effort increases sensitivity to reward and loss magnitude in the human brain. *Social Cognitive and Affective Neuroscience, 9*, 342–349. 10.1093/scan/nss147 PMC3980794 23202663

[CR25] Hulleman CS, Durik AM, Schweigert SB, Harackiewicz JM (2008). Task values, achievement goals, and interest: An integrative analysis. Journal of Educational Psychology.

[CR26] Hulleman CS, Schrager SM, Bodmann SM, Harackiewicz JM (2010). A meta-analytic review of achievement goal measures: Different labels for the same constructs or different constructs with similar labels?. Psychological Bulletin.

[CR27] Latham GP, Locke EA (1991). Self-regulation through goal setting. Organizational Behavior and Human Decision Processes.

[CR28] Linke J, Kirsch P, King AV, Gass A, Hennerici MG, Bongers A, Wessa M (2010). Motivational orientation modulates the neural response to reward. Neuroimage.

[CR29] Liu X, Hairston J, Schrier M, Fan J (2011). Common and distinct networks underlying reward valence and processing stages: A meta-analysis of functional neuroimaging studies. Neuroscience and Biobehavioral Reviews.

[CR30] Lopez-Paniagua D, Seger CA (2011). Interactions within and between Corticostriatal Loops during Component Processes of Category Learning. Journal of Cognitive Neuroscience.

[CR31] Luking, K. R., & Barch, D. M. (2014). Candy and the brain: Neural response to candy gains and losses. *Cognitive, Affective & Behavioral Neuroscience, 9*, 82–92. 10.3758/s13415-013-0156-8 PMC3915358 23519971

[CR32] Maddox WT, Ashby FG, Bohil CJ (2003). Delayed feedback effects on rule-based and information-integration category learning. Journal of Experimental Psychology: Learning, Memory, and Cognition.

[CR33] Mangels JA, Good C, Whiteman RC, Maniscalco B, Dweck CS (2011). Emotion blocks the path to learning under stereotype threat. Social Cognitive and Affective Neuroscience.

[CR34] McClure SM, Berns GS, Montague PR (2003). Temporal prediction errors in a passive learning task activate human striatum. Neuron.

[CR35] O’Doherty J, Dayan P, Schultz J, Deichmann R, Friston K, Dolan RJ (2004). Dissociable roles of ventral and dorsal striatum in instrumental conditioning. Science.

[CR36] O’Doherty JP (2004). Reward representations and reward-related learning in the human brain: Insights from neuroimaging. Current Opinion in Neurobiology.

[CR37] Pessiglione M, Seymour B, Flandin G, Dolan RJ, Frith CD (2006). Dopamine-dependent prediction errors underpin reward-seeking behaviour in humans. Nature.

[CR38] Peters J, Buchel C (2010). Neural representations of subjective reward value. Behavioural Brain Research.

[CR39] Poldrack RA, Clark J, Pare-Blagoev EJ, Shohamy D, Creso Moyano J, Myers C, Gluck MA (2001). Interactive memory systems in the human brain. Nature.

[CR40] Robbins TW, Everitt BJ (1996). Neurobehavioural mechanisms of reward and motivation. Current Opinion in Neurobiology.

[CR41] Rossion B, Kung C, Tarr MJ (2004). Visual expertise with nonface objects leads to competition with the early perceptual processing of faces in the human occipito-temporal cortex. Proceedings of the National Academy of Sciences.

[CR42] Satterthwaite TD, Ruparel K, Loughead J, Elliott MA, Gerraty RT, Calkins ME, Wolf DH (2012). Being right is its own reward: Load and performance related ventral striatum activation to correct responses during a working memory task in youth. Neuroimage.

[CR43] Schönberg T, Daw ND, Joel D, O’Doherty JP (2007). Reinforcement learning signals in the human striatum distinguish learners from nonlearners during reward-based decision making. Journal of Neuroscience.

[CR44] Schultz W, Dickinson A (2000). Neuronal coding of prediction errors. Annual Review of Neuroscience.

[CR45] Schultz W (2010). Subjective neuronal coding of reward: Temporal value discounting and risk. The European Journal of Neuroscience.

[CR46] Shohamy D (2011). Learning and motivation in the human striatum. Current Opinion in Neurobiology.

[CR47] Shohamy D, Myers CE, Grossman S, Sage J, Gluck MA, Poldrack RA (2004). Cortico-striatal contributions to feedback-based learning: Converging data from neuroimaging and neuropsychology. Brain.

[CR48] Spielberg JM, Miller GA, Warren SL, Engels AS, Crocker LD, Sutton BP, Heller W (2012). Trait motivation moderates neural activation associated with goal pursuit. Cognitive, Affective & Behavioral Neuroscience.

[CR49] Talairach J, Tournoux P (1988). Co-Planar stereotaxic Atlas of the human brain.

[CR50] Tricomi E, Delgado MR, McCandliss BD, McClelland JL, Fiez JA (2006). Performance feedback drives caudate activation in a phonological learning task. Journal of Cognitive Neuroscience.

[CR51] Tricomi E, Rangel A, Camerer CF, O’Doherty JP (2010). Neural evidence for inequality-averse social preferences. Nature.

[CR52] Wigfield A, Eccles J (2000). Expectancy-value theory of achievement motivation. Contemporary Educational Psychology.

[CR53] Zink CF, Pagnoni G, Martin ME, Dhamala M, Berns GS (2003). Human striatal response to salient nonrewarding stimuli. The Journal of Neuroscience.

