# Supplementary material for: Goals and task difficulty expectations modulate striatal responses to feedback
Source: Cogn Affect Behav Neurosci. 2014 Mar 18;14(2):610–20. doi: 10.3758/s13415-014-0269-8 (PMC4072914; doi:10.3758/s13415-014-0269-8)
Supplement: Supplementary file 1 — (PDF 128 kb) [file 13415_2014_269_MOESM1_ESM.pdf]

Supplemental Methods: Whole-brain Analyses:

We performed a whole-brain ANOVA to identify main effects of labeled difficulty ("EASY" or "HARD"), actual difficulty (low or high), and feedback valence. To explore the direction of the main effects, whole-brain contrasts were used to detect differences in brain responses to positive and negative feedback under different task difficulty expectations (positive versus negative when labeled "EASY," positive versus negative when labeled "HARD"). We additionally conducted a whole-brain Analysis of Covariance (ANCOVA) to explore relationships between normative goals and the effect of expectations on the pattern of feedback activation.

Clusters of voxels identified by the feedback-onset GLM analysis at an uncorrected statistical threshold of $p < 0.001$ were subjected to the cluster-level statistical threshold estimator in BrainVoyager, which provided a contiguity threshold for each contrast to produce a cluster level false positive alpha rate of 0.05.

Supplemental Results

Supplemental Table 1. Brain regions showing a main effect of feedback valence ($p < 0.05$, cluster threshold corrected).

Region of Activation	BA	Size (# voxels)	Peak Talairach Coordinates (*x, y, z*)			Maximum *F* Value
Positive feedback > Negative Feedback						
Cerebellum, posterior lobe, declive (R)		31022	38	-65	-18	45.29
Middle Frontal Gyrus (R)	46	742	53	40	18	28.92
Striatum (R)						
Putamen (ventral, anterior) (R)		13528	20	7	-6	93.18
**Putamen (dorsal)(R)*			*26*	*-5*	*12*	*42.64*
**Globus Pallidus(R)*			*26*	*-17*	*3*	*44.39*
Superior Parietal Lobule (BA 7)(R)	7	349	26	-68	42	20.40
Precuneus (BA 7)(R)	7	351	20	-56	51	21.23
Paracentral Lobule (BA 5)(R)	5	1995	5	-38	54	62.64
Posterior Temporal & Occipital Cortex (L)						
Occipital lobe, fusiform gyrus (BA 19) (L)	19	33659	-43	-71	-12	52.36
**Occipital lobe, lingual gyrus (L)*	19		*-31*	*-71*	*6*	*43.61*
**Temporal lobe, fusiform gyrus*	37		*-46*	*-38*	*-15*	*33.66*
Striatum (L)						
Putamen (ventral, posterior) (L)		14176	-28	-11	-6	61.32
***Caudate Body (L)*			*-21*	*4*	*21*	*27.23*
White matter (parietal/frontal lobe sub-gyral) (L)		354	-28	-29	30	26.34
Negative Feedback > Positive Feedback						
No regions exhibited greater feedback activation for negative than for positive feedback						

BA, Brodmann Area.
*To better identify the separate brain areas encompassed within the larger clusters, the threshold was increased until the larger clusters separated into smaller ones and those peaks are also reported.

Region of Activation	BA	Size (# voxels)	Peak Talairach Coordinates (x, y, z)			Maximum F Value
High Difficulty > Low Difficulty						
Middle Frontal Gyrus (R)	8	148	44	19	42	20.24
Insula (R)		113	35	10	6	23.04
Cerebellum, anterior lobe, culmen (R)		154	20	-23	-27	26.11
Thalamus (R)		161	5	-20	18	37.29
Inferior Frontal Gyrus (L)	11	66	-10	37	-18	30.72
Low Difficulty > High Difficulty						
No regions exhibited greater feedback activation during low difficulty blocks						

BA, Brodmann area.

Supplemental Table 3. Brain regions showing a main effect of labeled difficulty ($p < 0.05$, cluster threshold corrected).

Region of Activation	BA	Size (# voxels)	Peak Talairach Coordinates (x, y, z)			Maximum F Value
"HARD" > "EASY"						
Inferior Frontal Gyrus (R)	47	164	-34	25	-15	22.024
"EASY" > "HARD"						
No regions exhibited greater feedback activation during "EASY"-labeled blocks						

BA, Brodmann area.

Supplemental Table 4. Brain regions showing an interaction between feedback valence and difficulty label ($p < 0.05$, cluster threshold corrected).

Region of Activation	BA	Size (# voxels)	Peak Talairach Coordinates (x, y, z)			Maximum F Value
Middle Temporal Gyrus (R)	21	612	56	-20	-3	34.04
Inferior Temporal Gyrus (R)	20	59	53	-47	-9	23.89
Precuneus (L)	31	85	-7	-62	23	21.55
Superior Parietal Lobule (L)	7	98	-28	-47	57	23.17
Insula (L)	13	145	-37	-23	5	32.39

BA, Brodmann Area.

Region of Activation	BA	Size (# voxels)	Peak Talairach Coordinates (*x, y, z*)			Maximum *F* Value
High difficulty > Low difficulty						
Superior Frontal Gyrus	9	219	29	55	31	5.601711
Putamen		129	20	16	-3	4.558174
Lingual Gyrus	18	211	11	-68	3	4.764969
Thalamus		185	5	-20	18	6.096312
Anterior Cerebellum (Culmen)		1166	-22	-41	-15	6.724807
Insula	13	415	-37	13	15	4.805342

BA, Brodmann Area.

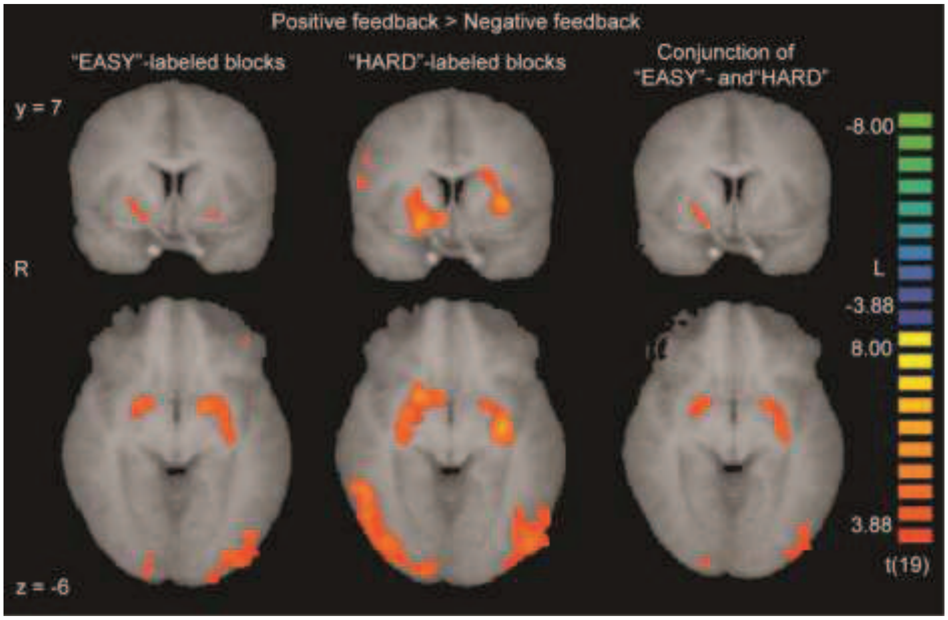

Supplemental Figure 1. Valence sensitivity in "EASY"- and "HARD"-labeled blocks. Positive > Negative feedback contrast activated the striatum in both "EASY"-labeled blocks (shown at left, $p < 0.05$, cluster threshold corrected; max $t = 7.07$) and "HARD"-labeled blocks (shown at center, $p < 0.05$, cluster threshold corrected; max $t = 6.80$), including the ventral striatum, putamen, and the body of the caudate nucleus. Posterior cortical regions including the fusiform and middle occipital gyri were also sensitive to feedback valence, as well as smaller regions in DLPFC, precuneus, and the paracentral lobule. Conjunction of "EASY" and "HARD" blocks is shown at right ($p < 0.05$, cluster threshold corrected).

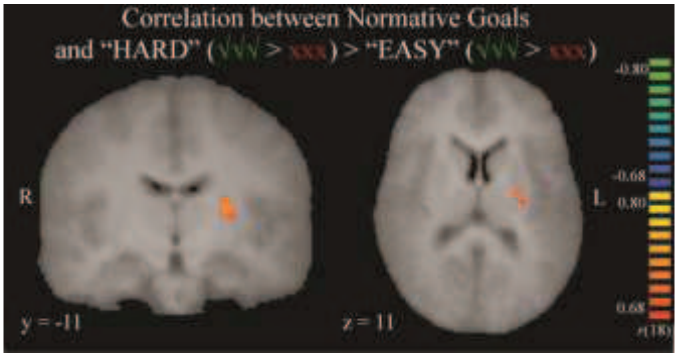

Supplemental Figure 2. Correlation between normative goals and expectation by valence interaction. In the dorsal putamen, normative goals were significantly positively correlated with the difference in "HARD" positive > negative feedback processing versus "EASY" positive > negative feedback processing (peak $r(18) = .75$, $p < 0.05$ corrected).
